# Geriatric drug trials on solid tumor are scarce worldwide

**DOI:** 10.3389/fmed.2023.1063648

**Published:** 2023-02-06

**Authors:** Huiyao Huang, Dandan Cui, Ye Leng, Kaiting Zhang, Anqi Yu, Yanting Wang, Hanli Wu, Yu Tang, Feng Yu, Ning Li

**Affiliations:** ^1^Department of Clinical Trials Center, National Cancer Center, Cancer Hospital, Chinese Academy of Medical Sciences and Peking Union Medical College, Beijing, China; ^2^Department of Clinical Trials Center, National Cancer Center/National Clinical Research Center for Cancer/Hebei Cancer Hospital, Langfang, China; ^3^School of Basic Medicine and Clinical Pharmacy, China Pharmaceutical University, Nanjing, Jiangsu, China; ^4^Phase I Clinical Research Center, Huashan Hospital, Fudan University, Shanghai, China; ^5^Department of Pharmacy National Cancer Center/National Clinical Research Center for Cancer/Cancer Hospital, Chinese Academy of Medical Sciences and Peking Union Medical College, Beijing, China; ^6^Department of Kidney Internal Medicine, Yidu Central Hospital of Weifang, Weifang, Shandong, China

**Keywords:** neoplasms, clinical trial, elderly patients, temporal trend, ageism

## Abstract

**Background:**

Conducting geriatric trials is the most feasible way to address the vast underrepresentation of older adults in clinical trials of cancer therapies. This study is a globally comprehensive examination of geriatric trials for solid tumor worldwide over the last decade.

**Methods:**

Up-to-date information on cancer drug trials in older adults aged over 59 years from the beginning of 2012 to the end of 2021 was collected from Trialtrove and Pharmaprojects. The number of identified trials was the dependent variable and corresponding analysis was conducted from the perspective of time trend, *status quo* and comparisons by region and country, sponsor type and cancer type, study status and phase.

**Results:**

A total of 292 geriatric cancer drug trials were identified, of which 287 were single-region studies, 219 were initiated by academic groups, and 55 (18.8%) were terminated. Decreasing trends in the annual number of all trials (−9.2% per year) and the annual number of trials by academic groups (−9.4%) were observed over time. Of the geriatric trials, 183 were conducted in Asia; this number was significantly higher than that in Europe (74), North America (37), Oceania (4), and South America (1). Similar difference was found in participation rate in trials by academic groups ranging from 71.7% in Asia to 0.5% in South America. Of the trials, 19 and 97 were initiated before drug and indication approval, respectively, and the remaining 176 were initiated after indication approval. Phase II trials accounted for the highest proportion of trials (213, 72.9%), while phase I trials accounted for the lowest proportion (14, 4.8%). Trials by academic groups had a higher termination rate (21.5% vs. 11.0%) and fewer were phase IV trials (8.2% vs. 21.9%). Treatment was explored for 16 different cancers, with lung, colorectal and breast cancers being the most common.

**Conclusion:**

Geriatric trials of solid tumor drugs are scarce and partially prematurely terminated. Moreover, the number of geriatric trials has decreased and differs according to region. Global guidance and regulatory supervision are needed to facilitate the acquisition of adequate evidence on drug risk-benefit profiles in older adults, and thus to achieve high-quality care and safe medication.

## Introduction

Older adults represent an increasing proportion of individuals with cancer worldwide (1). However, older adults are vastly underrepresented among registered trials of new cancer therapies; this population thus misses out on care improvements stemming from clinical research and lacks adequate evidence on drug efficacy and safety (2). More importantly, older cancer patients are a heterogeneous group, and evidence from younger patients regarding not only treatment safety and efficacy but also treatment goals (longer survival vs. better quality of life) may not be generalizable to older patients (3). Therefore, available evidence in the older population is essential for delivering high-quality care and safe medication.

Regulatory agencies have expressed concern and urged actions to reduce or eliminate inequities in evidence regarding cancer treatment of elderly individuals (4, 5). The Food and Drug Administration (FDA) issued several guidelines for the cancer drug industry, calling for the inclusion of older adults in clinical trials and recommending the addition of geriatric use information to labels (6, 7). Although upper-age restrictions have decreased among the enrollment criteria of cancer trials conducted in the United States, participation and representation of elderly individuals remain persistent challenges (8, 9). The reasons for the difficulties in accruing older patients are complex and multifaceted, including biological, social and cultural factors (10).

In addition to broadening the age eligibility criteria in pivotal trials, the most important recommendation for increasing the data available for older adults with cancer is to conduct separate studies in elderly individuals before or after drug approval (8). We believe that ensuring adequate data collection and evidence regarding drug safety and efficacy among older adults during drug development is an ethical imperative. Therefore, we conducted a comprehensive examination of cancer drug trials in elderly individuals among various regions. The primary goal of this study was to determine the overall status and trends of all geriatric drug trials on solid tumor during the last decade, to find out whether the industry has paid enough attention to the collection of adequate risk-benefit evidence on drug safety and efficacy in older patients. The secondary goal was to explore the potential differences of identified trials according to sponsor type, including in the region, development timing, trial scope, phase, status and outcome.

## Materials and methods

### Data source and study sample

Up-to-date information on cancer drug trials with elderly patients worldwide was retrieved from Trialtrove and Pharmaprojects, databases developed by an international research group providing digital services and academic knowledge (Informa Intelligence, London, UK) (11, 12). The two databases are known as the world’s most comprehensive, reliable and trusted source of pharmaceutical clinical trial data and drug development intelligence. Publicly accessible data from Trialtrove include both scientific and management items. The scientific items are mainly related to trial design, such as the title, tested drug, cancer type, therapeutic line, age range, sample size, treatment plan, study region and country, study phase, inclusion and exclusion criteria, and primary and secondary endpoints. The management items include trial ID, sponsor name, start date, primary completion date, study status, enrollment, trial outcome, etc.

We used therapeutic area, start date, and minimum patient age to identify relevant cancer drug trials in Trialtrove. Eligible trials were identified by the following inclusion criteria: (1) involving therapeutic medications for solid tumor; (2) start date ranging from 1 January 2012 to 31 December 2021; (3) minimum patient age greater than 59 years; and (4) primary endpoints of safety or efficacy. Trials that did not adhere to the above criteria were excluded; for example, hematology trials, trials with quality of life as the primary endpoint, and trials lacking a specified start date or minimum patient age were excluded. Overall, 292 unique trials were found to be eligible among the 612 initially identified trials.

Data extraction was performed to collect information on sponsor type (initiated by academic institutions or (co)initiated by companies), study scope (multiregional study or not), study stage (before approval, before indication approval or after indication approval), and minimum age group (60–64, 65–69, 70–74, or ≥75 years). To acquire information on the study stage, the Pharmprojects database was individually searched for the clinical development stage of the tested drug in the trial country at the start date for the specific cancer type. The study stage of the included trials was then classified accordingly.

### Study endpoints

The number of cancer drug trials involving elderly patients was the primary endpoint of our analysis. Our analysis was performed from three main perspectives: status (by region and country, study stage and cancer type), annual trends of trials (from 2011 to 2021), and sponsor type. Additionally, to further explore sponsor type, we performed subgroup comparisons among regions; study scope, stage, phase, and status; and minimum age group.

### Statistical analysis

SAS version 9.4 (SAS Institute, Cary, NC, US) was used for statistical analysis. The annual trends in the number of trials (overall and by sponsor type) were analyzed using a simple regression model. The annual number of trials was considered the dependent variable, and the time period was considered the independent variable. The coefficient of determination (*R*^2^) was used as a measure of model performance. In the descriptive analysis, qualitative variables are expressed as the number and percent (*n*, %). The χ^2^ test was used for subgroup comparisons between trials sponsored by academic groups and (co)initiated by companies as well as subgroup comparisons of different regions. A two-tailed *P* value of less than 0.05 was deemed significant.

## Results

### Temporal trends of initiated trials

**FIGURE 1:**
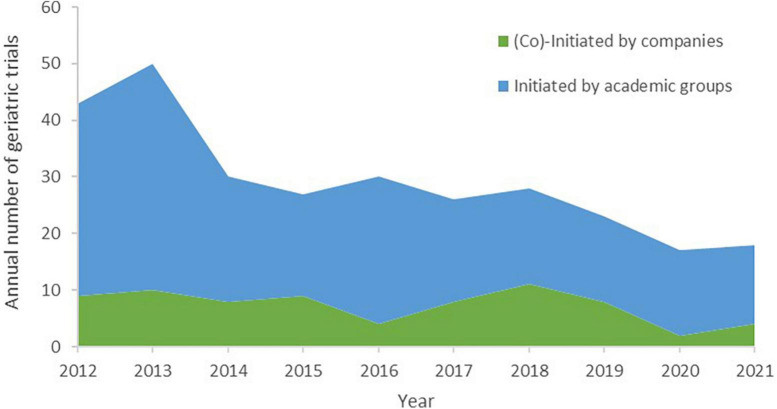
Annual number of initiated geriatric drug trials on solid tumor by sponsor type, 2012–2021.

From 2012 to 2021, a total of 292 drug trials on solid tumor in elderly individuals were identified, almost all of which were single-region studies. The minimum eligible age was mostly in the range of 71–74 years (43.2%), followed by ≥75 years (30.1%) and 66–70 years (19.5%); the minimum eligible age was distributed differently according to sponsor type (χ^2^ = 16.84, *P* = 0.0008, Table 1). A total of 222 (76.0%) trials were initiated by academic groups, 19 trials were initiated by companies, and 54 trials were coinitiated by academic groups and companies (Table 2). Decreasing trends over the last 10 years were observed regarding the annual numbers of all trials and trials initiated by academic groups on solid tumor in older patients, with average declines of 9.2% (*F* = 26.66, *R*2 = 0.769, *P* = 0.0008) and 9.4% (*F* = 18.08, *R*2 = 0.693, *P* = 0.0027), respectively. Trials (co)initiated by companies did not exhibit increasing or decreasing trends (*F* = 3.79, *R*2 = 0.321, *P* = 0.0875) (Figure 1).

**TABLE 1:** Subgroup comparison of initiated geriatric drug trials on solid tumor by sponsor type.

Region	Total				Sponsor type			
			(Co)-initiated by companies		Initiated by academic groups		Statistics	*P* value
Region							36.61	<0.0001
Oceania	4	1.40%	2	2.70%	2	0.90%		
South America	1	0.30%	0	0.00%	1	0.50%		
North America	37	12.70%	12	16.40%	25	11.40%		
Europe	74	25.30%	35	47.90%	39	17.80%		
Asia	183	62.70%	26	35.60%	157	71.70%		
Multiregional study							0.07	0.7945
Yes	5	1.70%	2	2.70%	3	1.40%		
No	287	98.30%	71	97.30%	216	98.60%		
Minimum age group							16.84	0.0008
60-64	21	7.20%	8	11.00%	13	5.90%		
66-70	57	19.50%	14	19.20%	43	19.60%		
71-74	126	43.20%	42	57.50%	84	38.40%		
>=75	88	30.10%	9	12.30%	79	36.10%		
Development timing							5.47	0.0649
Post indication approval	176	60.30%	42	57.50%	134	61.20%		
Before indication approval	97	33.20%	22	30.10%	75	34.20%		
Before drug approval	19	6.50%	9	12.30%	10	4.60%		
Study phase		0.00%						
I	14	4.80%	5	6.80%	9	4.10%	11.52	0.0092
II	213	72.90%	46	63.00%	167	76.30%		
III	31	10.60%	6	8.20%	25	11.40%		
IV	34	11.60%	16	21.90%	18	8.20%		
Drug type							17.8	<0.0001
Toxic drugs	156	53.40%	24	32.90%	132	60.30%		
Targeted drugs	84	28.80%	31	42.50%	53	24.20%		
Immune drugs	28	9.60%	9	12.30%	19	8.70%		
Ennocrine drugs	16	5.50%	5	6.80%	11	5.00%		
Other drugs	8	2.70%	4	5.50%	4	1.80%		
Treatment line							3.15	0.2069
Neoadjuvant or Adjuvant	42	14.40%	6	8.20%	36	16.40%		
First line	186	63.70%	51	69.90%	135	61.60%		
Second line or later	43	14.70%	11	15.10%	32	14.60%		
Unknown	21	7.20%	5	6.80%	16	7.30%		
Study status		0.00%					4.61	0.2028
Planned	7	2.40%	2	2.70%	5	2.30%		
Ongoing	100	34.20%	30	41.10%	70	32.00%		
Completed	131	44.90%	34	46.60%	97	44.30%		
Terminated	55	18.80%	8	11.00%	47	21.50%		
Terminated							3.95	0.0469
Yes	55	18.80%	8	11.00%	47	21.50%		
No	237	81.20%	65	89.00%	172	78.50%		
Ongoing							2.03	0.1544
Yes	100	34.20%	30	41.10%	70	32.00%		
No	192	65.80%	43	58.90%	149	68.00%		
Completed study outcome		0.00%					1.82	0.1769
Unknown	68	23.30%	14	41.20%	54	60.00%		
Positive outcome	56	19.20%	17	50.00%	39	43.30%		
Negative outcome	7	2.40%	3	8.80%	4	4.40%		

**TABLE 2:** Annual number of initiated geriatric drug trials on solid tumor by sponsor type, 2012–2021.

Year	(Co)-initiated by companies		Initiated by academic groups		Total
	No	%	No	%	
2012	9	20.9	34	79.1	43
2013	10	20.0	40	80.0	50
2014	8	26.7	22	73.3	30
2015	9	33.3	18	66.7	27
2016	4	13.3	26	86.7	30
2017	8	30.8	18	69.2	26
2018	11	39.3	17	60.7	28
2019	8	34.8	15	65.2	23
2020	2	11.8	15	88.2	17
2021	4	22.2	14	77.8	18
Total	73	25.0	222	76.0	292

### Geographical distribution of geriatric trials

**FIGURE 2:**
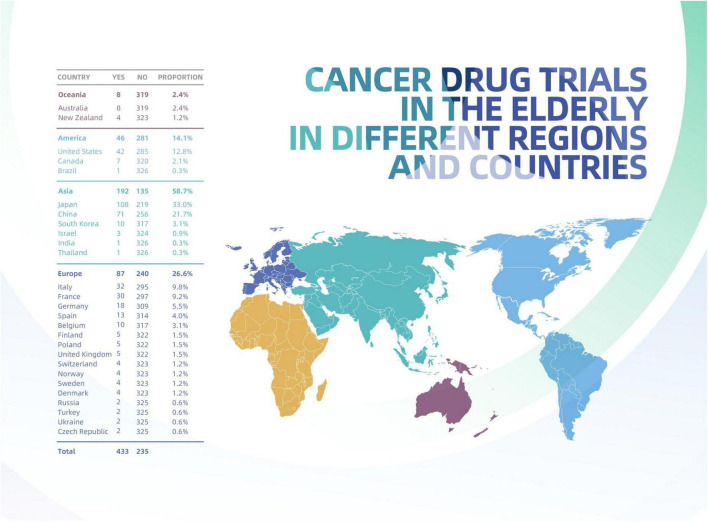
Geriatric drug trials on solid tumor by continent and country.

The number of included trials on solid tumor in elderly individuals varied by region and country, as shown in Figure 2. Most trials were located in Asia (183, 61.6%), followed by Europe (74, 24.9%); the fewest trials occurred in South America (1, 0.3%) and Oceania (4, 1.3%). The proportion of geriatric trials initiated by academic groups was significantly higher in Asia (71.7%) than in Europe (17.8%), North America (11.4%), Oceania (0.9%), and South America (0.5%), all *P* values < 0.0001 (Supplementay Appendix 1). The proportion of geriatric trials (co)initiated by companies was highest in Europe (47.9%), followed by Asia (35.6%) (Table 1).

Although Europe contributed only moderate numbers of trials, this region included the most countries contributing at least one geriatric trial to the present study, followed by Asia. At the country level, Japan (113) contributed most of the total trials included (38.0%), followed by China (64, 21.5%) and the United States (32, 10.8%).

### Clinical development stage and study phase of geriatric trials

Regarding drug development stage among geriatric trials on solid tumor, most studies were initiated after indication approval (176, 60.3%), followed by those initiated after drug approval and before indication approval (97, 33.2%), and those initiated before drug approval (19, 6.5%). Regarding study phase, phase II trials accounted for the highest proportion of included trials (213, 72.9%), while phase I trials accounted for the lowest proportion of include trials (14, 4.8%). No difference in clinical development stage was found according to sponsor type (χ^2^ = 5.47, *P* = 0.0649); however, sponsor type influenced study phase. Specifically, a slightly higher proportion of phase IV trials were (co)initiated by companies and academic groups (χ^2^ = 11.52, *P* = 0.0092).

**FIGURE 3:**
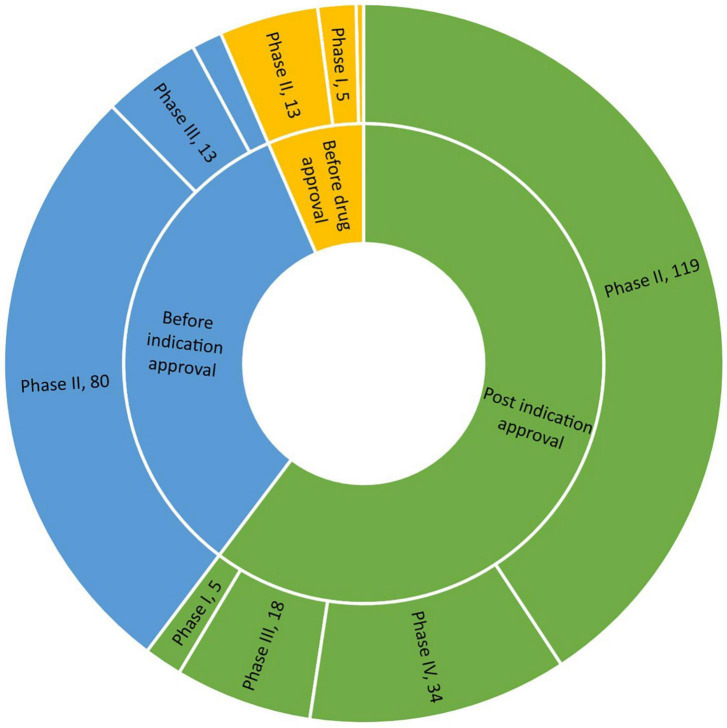
Development timing and study phase of initiated geriatric drug trials on solid tumors.

Next, we examined the distribution of geriatric trials according to clinical development stage and study phase. Phase I trials were relatively evenly distributed across clinical development stage, with 5 initiated before drug approval, 4 initiated before indication approval and 5 initiated after indication approval. This pattern differed from phase II trials. Most phase III trials with large sample sizes were seldom initiated before drug approval (1 out of 31 trials). Figure 3 presents more details on the distribution of geriatric drug trials on solid tumor according to clinical development stage and study phase.

### Cancer type and treatment line of geriatric trials

**FIGURE 4:**
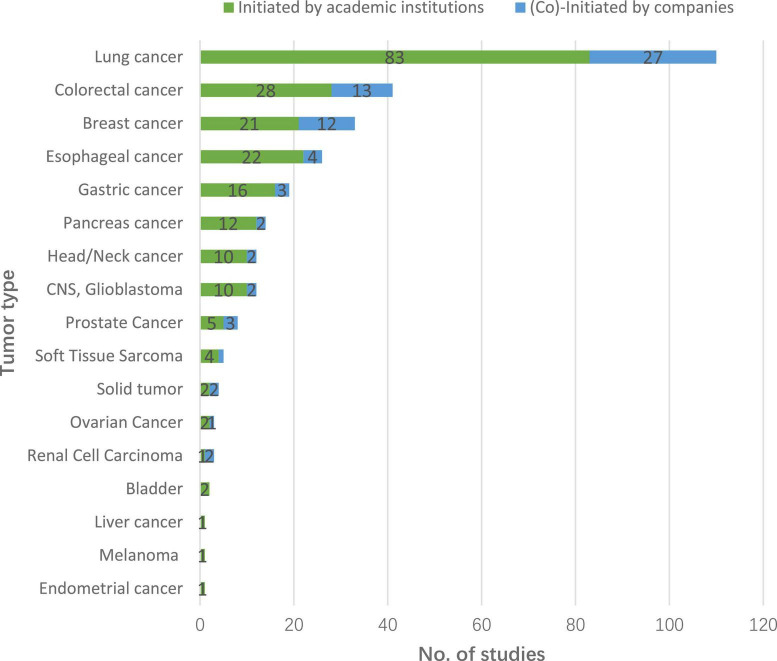
Cancer type distribution of initiated geriatric drug trials on solid tumors, 2012–2021.

Regarding cancer type, 4 trials were on unspecified solid tumor. The remaining 288 trials covered 16 different cancers. Lung cancer was the most common (110, 37.7%), followed by colorectal cancer (41, 14.0%), breast cancer (33, 11.3%), and esophageal cancer (26, 8.9%). More than half of the trials in older cancer patients (186, 63.7%) were tested in the context of first-line treatment, and 42 (14.4%) trials tested neoadjuvant or adjuvant therapies. Sponsor type did not appear to influence treatment line (χ^2^ = 3.15, *P* = 0.2069). Detailed information can be found in Figure 4 and Table 1.

### Study status and premature termination of geriatric trials

Among all 292 geriatric trials identified over the decade, 131 (44.9%) trials were completed by the end of August 2022, 100 (34.2%) trials were ongoing, 55 (18.8%) trials were terminated, and 7 trials were planned but not yet underway. Compared with geriatric trials sponsored by pharmaceutical companies, trials sponsored solely by academic groups were more likely to be prematurely terminated (11.0% vs. 21.5%, χ^2^ = 3.95, *P* = 0.0469).

## Discussion

This study pioneers to explore geriatric drug trials on solid tumor were at the global scale. We identified a total of 292 eligible studies and a decreasing trend over the past decade. This relatively low number may due to the lack of guidelines regarding adequate evidence on the drug risk-benefit profile among elderly individuals in pivotal trials as well as the lack of impact of inadequate evidence among older cancer patients on market size. Although it is common to report the distribution of participants aged over 65 years and over 75 years in pivotal studies on drug labels, indication approval does not impose any restrictions on data from elderly individuals (13). Thus, companies have low incentive to initiate geriatric trials; correspondingly, only 19 trials and 54 trials have been independently initiated and coinitiated by companies, respectively, in the past decade.

In terms of global guidance, calls for submission of data and stratified analyses related to the treatment of older adults should be issued, and regulatory agencies from different countries should exert maximum influence to supervise the analysis of drug risk-benefit ratios in the elderly population. If evidence from preapproval studies does not sufficiently address drug safety and efficacy in older adults, regulatory agencies should also consider requiring post marketing commitments from sponsors (3). Along with international guidance and legislative guarantees, educational interventions to increase sponsor investment in evidence-based strategies and public awareness regarding evidence-based medication are also important strategies to promote equity for older adults with cancer (8, 14).

Our analysis revealed that cancer drug trials in the elderly population, especially those initiated by academic groups, differed according to region: most trials were performed in Asia, followed by Europe, North America, Oceania and South America. This regional difference does not seem correlated with population aging among cancer patients, as the proportion of cancers patients over 65 years old is the lowest in Asia and the highest in Europe (1). Improving our understanding of the impact and determinants of cancer drug trials with elderly individuals is essential, particularly for cancer types and geographic regions where older people constitute the bulk of the cancer population.

We also explored study factors related to geriatric drug trials of solid tumor treatments, including clinical development stage, study phase and targeted age group. Less than 1 in 10 trials were initiated before drug approval, and few phase III trials were conducted in elderly individuals. This discrepancy could be attributed to multiple challenges in initiating such trials in older cancer patients, such as inadequate incentives, prolonged enrollment periods and high costs. Appropriate application of innovative approaches, such as adaptive designs, expansion cohorts, and hierarchical testing on different populations, in cancer drug trials with elderly patients could represent effective ways to address these issues and thus increase the possibility of exploring drug risk-benefit profiles in elderly individuals before drug approval (15). Cancer patients over 70 years of age were the age group most commonly enrolled in geriatric trials, with an aggregate ratio of 73.3%; this age limit also reflects the common older age limit in pivotal trials (16).

Our cross-sectional examination of cancer drug trials in the elderly population worldwide highlighted their poor performance; 18.8% of initiated trials were terminated, which was significantly higher than the termination rate of all cancer drug trials (8.08%) (17). This high termination rate could potentially be due to the three challenges mentioned above (inadequate incentives, prolonged enrollment periods, and high costs). Opportunities to leverage real-world data from electronic health databases to overcome the main challenges to conducting cancer drug trials with elderly patients have also been proposed in recent years (8, 18, 19).

Our analysis has several limitations. Potential errors in the database and missing data regarding minimum patient age are likely to lead to incomplete identification of study samples and potential bias. However, the scarcity of relevant trials and poor performance of initiated trials was observed across regions, regardless of sponsor type. Subgroup comparisons of identified trials should be interpreted with caution due to the limited number of included trials. Additionally, we did not examine reported outcomes of the identified trials, as we included all trials initiated by the end of 2021, and it takes additional time from initiation to reach the primary completion date and report outcomes.

## Conclusion

These findings suggest that geriatric drug trials for solid tumor are scarce, decreasing and diverging by region; most are academically funded and initiated after drug approval. The development of global guidance and regulatory supervision is warranted for the sake of high-quality care and safe medication and will facilitate adequate evidence regarding drug risk-benefit profiles in older adults.

## Data availability statement

The original contributions presented in this study are included in the article/Supplementary material, further inquiries can be directed to the corresponding author.

## Author contributions

HH, DC, and YL contributed to framework planning and draft writing, as well as data quality control, analysis, and interpretation. NL led the overall framework planning and data interpretation. KZ, AY, HW, YW, YT, and FY participated in framework planning and contributed to data interpretation. All the authors reviewed and revised the manuscript.
