# Supplementary material for: Geriatric drug trials on solid tumor are scarce worldwide
Source: Front Med (Lausanne). 2023 Feb 6;10:1063648. doi: 10.3389/fmed.2023.1063648 (PMC9939632; doi:10.3389/fmed.2023.1063648)
Supplement: Supplementary file 1 [file Table_1.docx]

| Subgroup comparisons of participation rate between regions: Overall and by sponsor type | | | | | | | | |
| --- | --- | --- | --- | --- | --- | --- | --- | --- |
| Subgroup comparison | All trials (N=292) | |  | Trials co-initiated by companies (N=73) | |  | Trials initiated by academic group (N=73) | |
|  | Statistics | *P* value |  | Statistics | *P* value |  | Statistics | *P* value |
| Asia-Europe | 82.56 | <0.0001 |  | 2.28 | 0.131 |  | 128.58 | <0.0001 |
| Asia-North America | 155.45 | <0.0001 |  | 6.97 | 0.0083 |  | 163.80 | <0.0001 |
| Asia-Oceania |  | <0.0001 |  |  | <0.0001 |  |  | <0.0001 |
| Asia-South America |  | <0.0001 |  |  | <0.0001 |  |  | <0.0001 |
| Europe-North America | 15.28 | <0.0001 |  | 16.70 | <0.0001 |  | 3.59 | 0.0582 |
| Europe-Oceania |  | <0.0001 |  | 39.42 | <0.0001 |  |  | <0.0001 |
| Europe-South America |  | <0.0001 |  |  | <0.0001 |  |  | <0.0001 |
| North America-Oceania |  | <0.0001 |  | 7.90 | 0.0049 |  |  | <0.0001 |
| North America-South America |  | <0.0001 |  |  | 0.0003 |  |  | <0.0001 |
| South America-Oceania |  | 0.3729 |  |  | 0.4966 |  |  | 1.0000 |
